# Research journey of respirasome

**DOI:** 10.1007/s13238-019-00681-x

**Published:** 2020-01-09

**Authors:** Meng Wu, Jinke Gu, Shuai Zong, Runyu Guo, Tianya Liu, Maojun Yang

**Affiliations:** 1 Ministry of Education Key Laboratory of Protein Science, Tsinghua-Peking Joint Center for Life Sciences, Beijing Advanced Innovation Center for Structural Biology, School of Life Sciences, Tsinghua University, Beijing, 100084 China; 2 School of Pharmacy, Tongji Medical College, Huazhong University of Science and Technology, Wuhan, 430030 China

**Keywords:** electron transport chain, supercomplex organization, cellular respiration, structure of respirasome, cryo-EM, megacomplex

## Abstract

Respirasome, as a vital part of the oxidative phosphorylation system, undertakes the task of transferring electrons from the electron donors to oxygen and produces a proton concentration gradient across the inner mitochondrial membrane through the coupled translocation of protons. Copious research has been carried out on this lynchpin of respiration. From the discovery of individual respiratory complexes to the report of the high-resolution structure of mammalian respiratory supercomplex I_1_III_2_IV_1_, scientists have gradually uncovered the mysterious veil of the electron transport chain (ETC). With the discovery of the mammalian respiratory mega complex I_2_III_2_IV_2_, a new perspective emerges in the research field of the ETC. Behind these advances glitters the light of the revolution in both theory and technology. Here, we give a short review about how scientists ‘see’ the structure and the mechanism of respirasome from the macroscopic scale to the atomic scale during the past decades.

## Introduction

Cellular respiration refers to a process during which the organic substrates undergoes a series of redox reactions within cells to produce inorganic or small molecular organic substances, releasing energy and generating adenosine triphosphate (ATP) molecules. Aerobic respiration might be the central function of mitochondria in mammalian cells, so no wonder it is precisely regulated from various aspects in response to varying cell conditions. Therefore, the disorder of its function will lead to changes in cell physiology and induce a variety of diseases, including many kinds of widely concerned neurodegenerative and inflammatory diseases such as Alzheimer’s disease, Huntington’s disease, Parkinson’s disease, Friedreich’s ataxia and so on (Sherer et al., 2002; Zeviani and Di Donato, 2004; Schapira, 2006; Pieczenik and Neustadt, 2007; Scharfe et al., 2009; Duchen and Szabadkai, 2010; Lim et al., 2010; Lax et al., 2011; Bates et al., 2012).

The oxidative phosphorylation (OXPHOS) in eukaryotic cell is the final step of aerobic cellular respiration, which occurs on the mitochondrial inner membrane. It is the main pathway of aerobic biosynthesis of ATP (Lenaz et al., 2016; Letts and Sazanov, 2017; Lobo-Jarne and Ugalde, 2018). OXPHOS in mammals is carried out by five classes of protein complexes anchored on the inner membrane of mitochondria. These complexes are relatively independent in both structure and function, including complex I (NADH:ubiquinone oxidoreductase, CI), complex II (succinate:ubiquinone oxidoreductase, CII), complex III (cytochrome *bc*_1_ complex, CIII), complex IV (cytochrome c oxidase, CIV), and complex V (ATP synthase, CV) (Wharton and Tzagoloff, 1962; Green and Tzagoloff, 1966; Hatefi, 1985; Papa et al., 2012; Zong et al., 2018a; Zong et al., 2018b; Gu et al., 2019). Complex I–IV are also called respiratory chain complexes, or electron transport chain (ETC) complexes, because they only participate in the process of electron transportation and oxygen consumption.

OXPHOS in essense is the transfer of electrons along the ETC to the oxygen and the translocation of protons from the mitochondrial matrix (MM) into the intermembrane space (IMS), which forms the electrochemical gradient of proton. The ATP molecules are synthesized by CV, as protons diffuse back into the MM. During this process, CI, CIII and CIV act as proton pumps, while CII does not.

The transfer pathway of electrons in ETC could be described as such: CI oxidizes NADH to NAD^+^ and reduces ubiquinone-10 (Q) to ubiquinol-10 (QH_2_). CII could also reduce Q to QH_2_ by oxidizing succinate to fumarate. QH_2_ is reoxidized to Q by CIII, and cytochrome c (cyt c) is reduced in the meantime. CIV transfers the electrons from the reduced cytochrome c to O_2_ sequentially. The energy released in this process is used to generate ATP (Milenkovic et al., 2017; Hirst, 2018).

The organization of the respiratory chain complexes has been widely studied for decades. Many evidence show that the individual complexes could assemble into supercomplexes (I_1_III_2_IV_1–2_, III_2_IV_1–2_), or even megacomplex (I_2_III_2_IV_2_) (Schägger and Pfeiffer, 2000; Schagger and Pfeiffer, 2001; Bultema et al., 2009; Mileykovskaya et al., 2012; Greggio et al., 2017; Guo et al., 2017). These higher order complexes may be beneficial for maintaining the biochemical structure and increasing the physiological activity of the individual complexes, while improving the efficiency of electron transportation. A widely held opinion points out that the interaction between different complexes in the supercomplexe (SC) may be conducive to the possibility of the existence of the substrate channelling as functionally segmented CoQ pool. Supercomplexes could also reduce the production of the reactive oxygen species (ROS) by sequestrating reactive intermediates (Enriquez, 2016). These functionally active supramolecular structures are also known as respirasomes.

From biochemical experiments, to blue native polyacrylamide gel electrophoresis (BN-PAGE) analysis, from X-ray crystallography to cryo-electron microscopy (cryo-EM), advances in technology promote the development of how we understand the structure, the assembly, and the mechanism of the ETC complexes. Till today, the discussion is still far from ending. With the report of the high- resolution structures of mammalian respiratory supercomplex-I_1_III_2_IV_1_ (SCI_1_III_2_IV_1_) and the recognition of megacomplex-I_2_III_2_IV_2_ (MCI_2_III_2_IV_2_) by us and several other groups, more details are presented, leading to the proposal of some new viewpoints and even more considerable controversy (Gu et al., 2016; Letts et al., 2016; Sousa et al., 2016; Wu et al., 2016; Guo et al., 2017; Guo et al., 2018). In this paper, we will give a brief review of the history of the research on respirasome, combined with the development of technology, and introduce some lately reported results and heatedly debated arguments.

## Pre-structural age, their existence and their function

The study of the OXPHOS system has gone through a long history. After the instructive investigation of Otto Warburg who discovered Atmungsferment, which founded the enzymatic basis for respiration, tantalizing advances emerged to clarify the molecular mechanisms of the respiratory enzymes. The term oxidative phosphorylation was first proposed by Volodymyr Belitser in 1939, who measured the P/O ratios (a division of the rate of ATP production and oxygen consumption) of OXPHOS in minced and homogenized heart muscle and pigeon breast muscle, indicating the possibility that some intermediate redox reactions are coupled with phosphorylation. During 1900s to 1930s, active work on bioenergetics were done by spectral and chromatographic analysis and biochemical experiments (Kalckar, 1974, 1991). In 1955, Britton Chanc and G.R.Williams first raised the idea that the redox enzymes and prosthetic groups that are supposed to be responsible to electron transportation could assemble into larger complexes, which were then reconstituted as electron transfer system (Chance and Williams, 1955). Four functional complexes were purified and reconstructed by Hatefi et al., till 1962, termed CI-CIV (Hatefi et al., 1962). Together with the isolation of electron donors NADH and FAD and the identification of the roles of Q and cyt c, the ETC system gradually matured (Hill and Keilin, 1930; Crane et al., 1957; Enríquez, 2016). Other redox reactions providing electrons to reduce coenzyme Q such as dihydroorotate dehydrogenase (Evans and Guy, 2004), glycerol 3 phosphate dehydrogenase (Harding et al., 1975) and electron transport flavoprotein dehydrogenases (Bentinger et al., 2010; Alcázar-Fabra et al., 2016) are also found.

A question ensues, how does ETC drive ATP synthesis? The most commonly accepted theory was the chemiosmotic hypothesis raised by Peter D. Mitchell in 1961 (Mitchell, 1961). The work of Hatefi et al. provided support to the chemiosmotic hypothesis (Hatefi et al., 1962) because their purified ETC complexes showed enzymatic activities. Chemiosmotic hypothesis advocates for a reversible proton translocation ATPase system and oxido-reduction chain that could make use of the diffusion-driven force known as proton-motive force (PMF) generated by the electrochemical potential between both sides of the mitochondrial inner membrane to produce ATP. The interaction between ETC complexes and ATP synthase is unnecessary, while the battery like mitochondrial inner membrane stores the energy needed (Mitchell, 2011; Enríquez, 2016; Hirst, 2018). This is inconsistent with the previous idea that the respiratory components were rigidly coupled as a functional unit, which requires complicated regulation of stoichiometries at each step (Enríquez, 2016).

The process of how ETC complexes implement their functions is closely linked with another question: how do ETC complexes organize on the mitochondrial inner membrane? Three models have been proposed successively: the solid model, fluid model (also known as random diffusion model) and plasticity model. The solid model proposed by Keilin, Chance et al. regarded ETC catalytic complexes as solidly associated single units that can catalyze whole reaction pathways, with their electron carriers also enclosed in the pathways allowing for a better electron transporting efficiency (Keilin and Hartree, 1947; Chance and Williams, 1955; Chance et al., 1963; Blair, 1967; Lenaz and Genova, 2007; Acin-Perez et al., 2008). Whereas in the fluid model, individual ETC complexes and redox components move in diffusional motion constantly and independently in the membrane, and the electrons are transferred between the complexes through the free diffusion of Q and cyt c (Hackenbrock, 1977). The fluid model gained general recognition for decades and was consistent with the chemiosmotic hypothesis (Hatefi et al., 1962; Kroger and Klingenberg, 1973; Hochli and Hackenbrock, 1976; Hochli et al., 1985; Gupte and Hackenbrock, 1988). However, as time went on, neither of these two models could withstand all of the surfacing experimental evidence.

## Era of X-ray crystallography, an unremitting pursuit

Structural biology has been an important subject that enables people to understand the structure of biological molecules in great detail. Multiple methodologies derived from different mathematical and physical principles have been exploited to determine the spatial relationship of the atoms within biological molecules. The most widely implemented methods include nuclear magnetic resonance (NMR) spectroscopy, X-ray crystallography, neutron diffraction, cryo-EM, and spectroscopic techniques. X-ray crystallography has been the most powerful tool in resolving structures of macromolecules at atomic resolution since its birth over a century ago (Shi, 2014; Powell, 2017; Wang and Wang, 2017; Standfuss, 2019). More than 138,000 molecular structures, reported with this technique, have been deposited in the Protein Data Bank (PDB) since its establishment in 1971, endowing X-ray crystallography with the dominant role in structural biology. From the late 1960s, research on ETC has begun to incorporate structural biological techniques.

The first successful case of structural analysis of ETC complexes was the 2.8 Å crystal structures of CIV extracted from bacterial and bovine heart published in 1995 and 1996 (Iwata et al., 1995; Tsukihara et al., 1995, 1996). CIV in crystal structures existed as dimers with each monomer consisting of 13 different subunits. In contrast, we proved CIV is a 14-subunit monomer in native state recently (Zong et al., 2018b). In 1998, the precise locations of its cofactors, heme a, heme a_3_, Cu_A_ and Cu_B_ were identified (Brzezinski and Adelroth, 1998). In the year 2012, Eduardo Balsa and partners pointed out that NDUFA4 which was considered to be a subunit of CI was actually the subunit of CIV (Balsa et al., 2012).

It is now recognized that mammalian CIV is composed of three core subunits encoded by mitochondrial genes (COXI, COXII and COXIII) and eleven subunits encoded by nuclear genes. Cofactors heme a (containing Fe_a_), heme a_3_ (containing Fe_a3_) and Cu_B_ are located in subunit COXI, with heme a_3_ (Fe_a3_) and Cu_B_ forming a binuclear center. Cu_A_ is in subunit COXII. These four redox-active metal centers constitute an electron transport pathway. In each catalytic cycle, Cu_A_ accepts electrons donated from four reduced cyt c consecutively and transfer them to the active site through Fe_a_. Two protons are taken from MM with one electron transported to the Fe_a3_/Cu_B_ binuclear center. In total, eight protons are uptaken from MM, with four of them used to produce H_2_O and four pumped into IMS to form the proton gradient (Konstantinov et al., 1997; Brzezinski and Adelroth, 1998; Sousa et al., 2018). Three channels for proton transportation named D-, K-, and H-channel have been described (Wikstrom et al., 2015). The K- channel is responsible for the transportation of two protons consumed in the reduction of O_2_ to H_2_O from MM to the binuclear center for water formation. The D- channel conducts the other two protons consumed for the reduction of O_2_, and provide the pathway for the four protons pumped into the IMS (Konstantinov et al., 1997; Brzezinski and Adelroth, 1998). The H- channel consist of water cavities and polar residues, and is supposed to be functionally associated with a hydrogen- bond network linked to oxido-reduction of heme a (Yoshikawa et al., 1998; Yoshikawa and Shimada, 2015; Papa et al., 2018).

CIII was the second member to get its crystal structure in ETC complexes. The first complete structure of CIII was obtained in 1998 (Iwata et al., 1998). Iwata et al. purified CIV from bovine heart and reported its atomic structure as a symmetrical homodimer consisting of 22 subunits in total. However, we found CIII is actually an asymmetric 21-subunit dimer in 2018 (Zong et al., 2018a). The mammalian CIII monomer is composed of three highly conserved core subunits and eight supernumerary subunits. The core subunits include a mitochondrial encoded cytochrome b (cyt b) with heme b_L_, heme b_H_ and two distinct quinone-binding sites, a nuclear-encoded cytochrome c_1_ (cyt c_1_) with heme c_1_, and a Rieske iron-sulfur protein (ISP) with a [2Fe-2S] cluster (Yang and Trumpower, 1986). Two quinone-binding sites named Q_o_ and Q_i_ in cyt b locate on opposite sides of mitochondrial inner membrane (Xia et al., 1997; Pietras et al., 2016). A feature of CIII dimer is that each ISP subunit of a monomer spans two monomers with the transmembrane domain associating with one monomer while the soluble domain remains in the other monomer. It is still controversial whether the two monomers of CIII_2_ function cooperatively or independently.

Crystal structures of CII were first studied in prokaryote. Iverson and partners reported the 3.3 Å structure of *Escherichia coli* fumarate reductase (QFR) (Iverson et al., 1999). In 2003, Yankovskaya et al. reported the structure of CII (SQR) (Yankovskaya et al., 2003). The first mammalian CII crystal structure at a resolution of 2.4 Å was determined in 2005 with porcine heart (Sun et al., 2005). CII is composed of an FAD binding protein (flavoprotein,Fp), an iron-sulfur protein (Ip) and two membrane-anchor proteins (CybL and CybS). Fp and Ip form the hydrophilic head, while CybL and CybS form the hydrophobic arm. Three kinds of prosthetic groups, FAD, heme and iron-sulfur clusters, were recognized in CII, coupled with two Q-binding sites (Q_P_ and Q_D_). Herein, Fp contained the FAD cofactor, Ip contained three iron-sulfur clusters ([2Fe-2S], [4Fe-4S] and [3Fe-4S]), yet CybL and CybS each had a heme b (Cecchini, 2003; Bezawork-Geleta et al., 2017). The Q-binding sites were investigated by means of mutagenesis and kinetic analysis with inhibitors. Q_P_ site is proximal to the matrix side of inner mitochondrial membrane (IMM), and Q_D_ site is distal from the matrix. During the succinate oxidation reaction, two electrons are transferred from the falvin to reduce Q bound at Q_P_ via the iron-sulfur clusters [2Fe-2S], [4Fe-4S] and [3Fe-4S]. There is little categorical data explicating the role of the heme and Q_D_ site (Bezawork-Geleta et al., 2017; Sousa et al., 2018).

CI is the largest and most complicated protein complex in ETC and is vital to cellular metabolism. In many eubacteria, this type of enzyme is termed as NADH dehydrogenase-1 or NDH-1. The sodium-pumping NADH-quinone reductase (Na^+^-NQR) and the type II NAD(P)H dehydrogenase (NDH-II) are also members of this protein family (Melo et al., 2004; Barquera, 2014). In plants, many fungi and many bacteria, four so-called alternative NADH dehydrogenases are found, which do not couple the redox reaction to proton or sodium translocation (Kerscher, 2000; Brandt, 2006; Kerscher et al., 2008; Sousa et al., 2018). After its purification from bovine heart in 1962, the molecular structure of CI remained elusive for a long time. The architectures of CI were determined with electron microscopy at the early stage (Leonard et al., 1987; Hofhaus et al., 1991; Grigorieff, 1998; Peng et al., 2003; Radermacher et al., 2006). It was not until 2010 that scientists resolved the first crystal structure of the entire CI from *Y*. *lipolytica* at a resolution of 6.3 Å (Hunte et al., 2010). However, a complete atomic resolution structure obtained with X-ray crystallography is still lacking.

Structural analysis of CI in this era indicates that with a molecular mass of about 970 kDa, integral mammalian CI is composed of 45 subunits assembled into an L-shaped architecture. The minimal functional unit of mammalian CI comprises 14 subunits known as core subunits. Subunits ND1-ND6 and ND4L are encoded by mitochondrial genome and form the hydrophobic domain contained in the mitochondrial inner membrane. The other seven core subunits form the hydrophilic arm comprising a flavin mononucleotide (FMN) and eight iron–sulfur clusters as redox active prosthetic groups and extends into the MM. Up to 31 supernumerary subunits (include two NDUFAB1 subunits) are identified in the intact mammalian CI. These subunits play an important role in the assembly, stabilization and regulation of CI and fulfill the independent function of mitochondrial metabolism. In CI, two electrons are transferred from NADH to FMN and then to quinone via seven iron-sulfur clusters (N3, N1b, N4, N5, N6a, N6b, and N2). Cluster N2 is the direct reductant for quinone. Cluster N1a may play a role in preventing the excessive production of ROS (Sazanov et al., 2013; Friedrich, 2014; Sazanov, 2015). Four protons are translocated into the IMS during this process. Many hypothetical mechanisms have been proposed to clarify the coupling between electron and proton transfers. Evidence suggest that long-range conformational change may be related to this process. One or two-stroke mechanisms have been discussed. One-stroke mechanism offers a model in which four protons are translocated all at once, driven by the redox of one quinone molecule. The two-stroke model, on the other hand, proposes that two sequential one-electron Q reduction steps induce two conformational changes, each translocating two protons (Brandt, 2011; Efremov and Sazanov, 2012; Hirst, 2013).

Aside from CI, in various fungi, plants, and primitive cells, an alternative enzyme was found able to reduce Q, but lacking the proton pumping capacity. This alternative enzyme, NDH2 (NADH dehydrogenase type-II), was first identified in domestic yeast as a 50 kDa single-subunit membrane protein (Ohnishi et al., 1966), and was shown to be essential for the long-term survival of some pathogenic microorganisms (Biagini et al., 2012; Verner et al., 2013; Yano et al., 2014), like *P*. *falciparum*, *Toxoplasma gondi*, and *Mycobacterium tuberculosis*. In 2012, our group solved the X-ray structures of Ndi1 (NDH2 from yeast) in apo, NADH-, Q-, and NADH-Q-bound states and demonstrated that electron transfer in NDH2 requires two Q molecules and CTD of NDH2 mediates the homodimerization and membrane attachment (Feng et al., 2012). Later on, our group reported the X-ray structures of *Pf*NDH2 (NDH2 from *P*. *falciparum*) in its apo, NADH-, and RYL-552 (a new inhibitor)- bound states, unveiling the inhibiting mechanism of *Pf*NDH2 and providing accurate information for designing new anti-malarial drugs (Yang et al., 2017).

The X-ray crystallography studies of ETC complexes provide important structural information for the study of respiration. However, the biostructural research of ETC complexes is far from perfect. In the age of cryo-EM today, new exciting discoveries are emerging, helping build the edifice of respiratory system.

## Identification of respirasome and the plasticity model

The crystal structures of individual ETC complexes are gradually reported with the efforts of researchers. However, the investigation on the functional mechanism of ETC is still filled with dark clouds. The existence of higher-order organization of ETC complexes has been considered for a long time. The invention of BN-PAGE technology created a new climate to the debates. BN-PAGE can be used to isolate protein complexes and determine their native protein masses and oligomeric states. The first BN-PAGE analysis was carried out by Schägger and Pfeiffer in 2000 with digitonin-solubilized mitochondrial extracts from yeast and bovine, revealing the co-migration of respiratory complexes in sucrose gradient centrifugation and in gel (Schägger and Pfeiffer, 2000). This finding led to the revival of the solid model. The concept of respirasome was then proposed. Respirasome was originally considered to have a fixed composition as two copies of SCI_1_III_2_IV_4_ and one copy of SCIII_2_IV_4_ to consist with the overall 1:3:6 stoichiometric ratio of complexes I:III:IV (Schägger and Pfeiffer, 2000; Wittig et al., 2006a). However, these kinds of entities were not detected. Contrarily, superassemblies as I_1_III_2_, III_2_IV_1_ and free complexes were observed. Henceforth, although accompanied with significant skepticism, SCs from different sources such as yeast and other fungi, plants, vertebrates, and invertebrates were found by BN-PAGE with a variety of detergents (Eubel et al., 2004; Krause et al., 2004a; Krause et al., 2004b; Stroh et al., 2004; Krause, 2006; Reifschneider et al., 2006; Marques et al., 2007; Bultema et al., 2009). The co-migration on sucrose gradients also indicates the existence of SCs. Another convincing proof is the detection of SCs in the tomograms of cristae isolated from Podospora anserine (Davies et al., 2011). Recent subtomogram averaging results give a further confirmation of the ubiquitous existence of respirasomes (Davies et al., 2018). *In vitro* experiments suggest that the isolated respirasomes still possess the ability to transfer electrons from NADH to oxygen (Acin-Perez et al., 2008; Milenkovic et al., 2017). The stability of CI is dependent on CIII_2_ and CIV (Schagger et al., 2004; Stroh et al., 2004).

**Figure 1:**
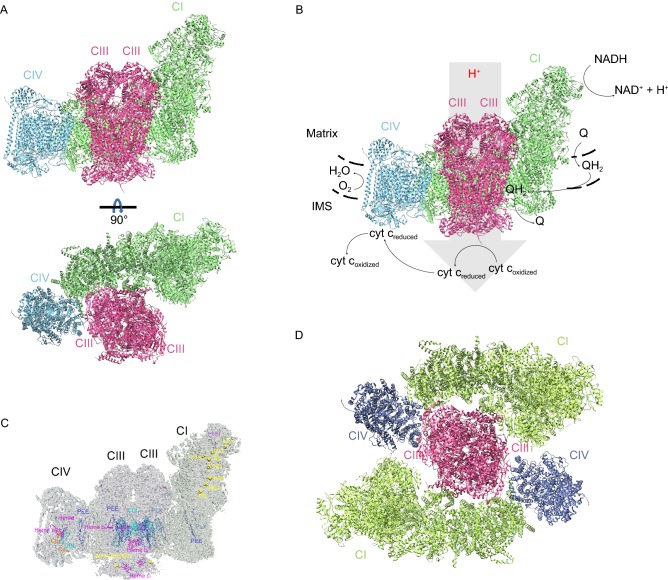
**Structures of Mammalian SCI**_**1**_**III**_**2**_**IV**_**1**_**and MC I**_**2**_**III**_**2**_**IV**_**2**_. (A) Cartoon representation of the cryo-EM structure of human SCI_1_III_2_IV_1_ (PDB ID: 5XTH) shown in two orientations. The side view along the inner membrane, and the top view perpendicular to the inner membrane. Individual CI, CIII, and CIV monomers are labeled with texts in the same colors with the represented structures, respectively. (B) Substrate translocation in SCI_1_III_2_IV_1_. The black dotted lines represent mitochondrial inner membrane. The grey arrow indicates the translocation of protons. The black arrows indicate changes of substrates before and after the redox reactions. (C) Cofactors in SCI_1_III_2_IV_1_. Different cofactors are shown in different colors. Hemes are shown as stick and ball model, FeS clusters and ions are shown as spheres, and phospholipid molecules and FMN are shown as lines. The colors of the label texts are the same with colors of the represented structures. (D) Cartoon representation of the structure of human MCI_2_III_2_IV_2_ (PDB ID: 5XTI)

The majority of CI is found to assemble into SCs with CIII and CIV, while CII tends to exist in a non-associated form in plant and mammalian mitochondria (Schagger and Pfeiffer, 2001). The most common forms of SCs include SCI_1_III_n_, SCI_1_III_2m_IV_n_ and SCIII_2m_IV_n_ (Maranzana et al., 2013). The most interesting one among which is SCI_1_III_2_IV_1_ (Fig. 1A), because this supercomplex contains all components required to accomplish the electron transportation, from NADH to oxygen together with cyt c and quinone, and is conserved in mammalian mitochondria (Schägger and Pfeiffer, 2000; Lenaz and Genova, 2012). Only a few cases hypothesize the association of CII.

The fact that both free and diversely superassembled respiratory complexes are able to partake in cellular respiration has spawned a proposal called plasticity model. This view suggests a dynamic situation where the respiratory complexes can exist and function in both free and diversely superassembled modes. The stoichiometries and stabilities of free and superassembled complexes may vary with cell types and physiological stimuli (Acin-Perez et al., 2008; Lenaz and Genova, 2012; Enríquez, 2016). This dynamic equilibrium state is supported by some indirect evidence (Gomez et al., 2009; Frenzel et al., 2010; Hofmann et al., 2012). Starvation may reduce the amount of SCI_1_III_2_IV_1_ in mice liver mitochondria (Lapuente-Brun et al., 2013). Hypoxia induces the dissociation of the SCI_1_III_2_IV_1_ into free CI and the SCIII_2_IV in potato, while the expression of alternative oxidase (AOX, a Q oxidase found in plants and fungi, but not found in most animals) can stable free CI without the presence of CIII and IV (Ramirez-Aguilar et al., 2011). The validity of the reversible dynamic association/dissociation between ETC complexes and supercomplexes remains unproven.

## Cryo-EM, old trees and new buds

The ability to purify SCs from mitochondria allows for their visualization and structural determination at low resolutions. As a promising technology gaining great attention in recent years, cryo-EM has come to exhibit its latent capacity in the field of structural biology. The advantages of cryo-EM are as follows: less sample requirement, no need for crystallization, less limitation on sample purity, and suitable for the structural analysis of biological molecules or complexes with larger molecular weight. However, 3D reconstruction using cryo-EM is by no means a newborn methodology.

In 1926, German physicist Hans Busch put forward the idea of using magnetic field generated in the short coil of a cathode ray tube to condense electron beam and use it for imaging. Based on this theory, Ruska, together with partners, developed the first EM in 1930s and was awarded the Nobel Prize in Physics in 1986 (Robinson, 1986).

The history of analyzing the structure of biological macromolecules by transmission electron microscopy can be traced back to 1960s. DeRosier and Klug published the first 3D EM structure of the tail of *bacteriophage T4* and formulated the general principles of 3D reconstruction tack using helical Fourier inversion method simultaneously (De Rosier and Klug, 1968). To avoid the radiation damage caused by the beam induced breakage of chemical bonds, Dubochet and colleagues developed the method of freezing the samples into a thin layer of amorphous ice (also referred to as vitreous ice) (Dubochet et al., 1981; Adrian et al., 1984; Lepault et al., 1987; Dubochet et al., 1988) based on the early study of Henderson and Unwin as well as that of Taylor and Glaeser (Taylor and Glaeser, 1974; Henderson and Unwin, 1975; Hayward and Glaeser, 1979; Bai et al., 2015). The samples are stored and imaged at the temperature of liquid nitrogen; hence, the naming “cryo-EM”. The vitrified samples anchor the molecules in a close-to-native environment, preserving the structural details of particles without introducing artifacts. Meanwhile, flash-frozen amorphous ice helps prevent the dehydration of biological samples in the vacuum during imaging.

**Figure 2:**
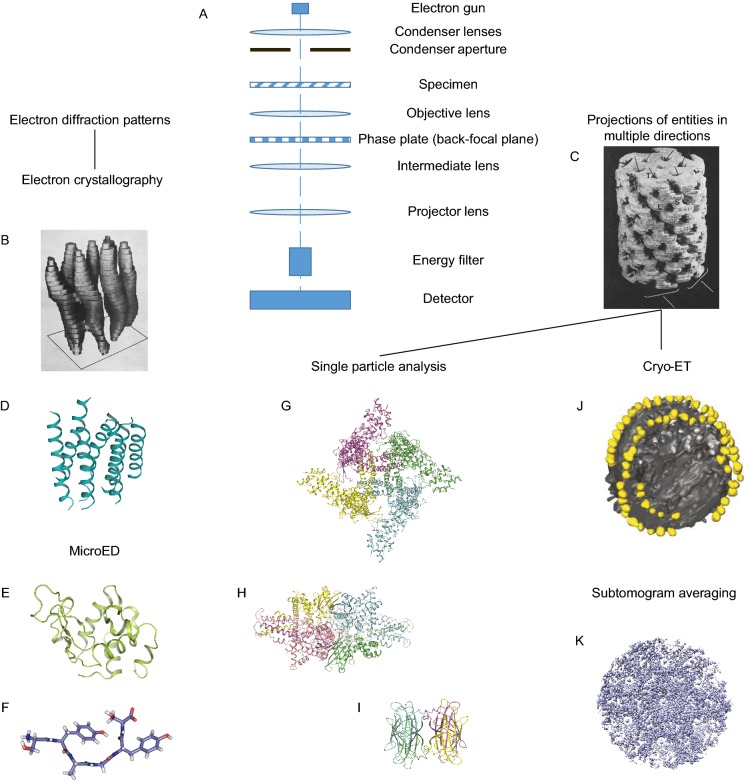
**Evolution of EM in Structural Biology.** (A) Schematic diagram of a transmission electron microscope (TEM) equipped with a phase plate, energy filter and direct electron detector. (B) The first map of bacteriorhodopsin determined by electron crystallography at a resolution of 7 Å (Henderson and Unwin, 1975). The image of the map is derived from the original paper. (C) The first map of the tail of Bacteriophage T4 obtained on the basis of Fourier inversion method. The image of the map is derived from the original paper. (D) First near-atomic-resolution biological macromolecular structure of bacteriorhodopsin obtained by electron crystallography (PDB ID: 1brd) (Henderson et al., 1990). (E) 2.9 Å structure of lysozyme (PDB ID: 3J4G), laying the first stone for the MicroED technique. (F) 0.7 Å structure of RNA-binding protein FUS (37–42) SYSGYS segment determined by MicroED (PDB ID: 5XSG) (Luo et al., 2018). (G) 3.3 Å structure of TRPV1 ion channel determined by single particle analysis method, representative near-atomic-resolution structure obtained with DDD and motion correction method (PDB ID: 3J5P) (Liao et al., 2013). (H) 1.8 Å structure of glutamate dehydrogenase (PDB ID: 5K12) (Merk et al., 2016). Published structure of highest resolution obtained with single particle analysis method. (I) 3.2 Å structure of 52 kDa biotin-bound streptavidin (PDB ID: 6J6J) (Fan et al., 2019). Smallest integral biological macromolecular structure determined at near-atomic resolution. (J) Tomographic volumes of respiratory CV dimer ribbons (Strauss et al., 2008). The image of the map is derived from the original paper. (K) 3.4 Å density map of HIV-1 dMACANC VLPs (EMDB ID: emd_3782) (Turonova et al., 2017), indicating a successful trial of subtomogram averaging and 3D-CTF algorithm

Three main branches have been derived from the cryo-EM technology: 2/3D electron crystallography, single particle 3D reconstruction, and cryo-electron tomography (cryo-ET). Electron crystallography is the first branch to reach near-atomic and atomic resolutions. However, because of the relatively harsher conditions for sample preparation and screening, the application of this method has been restricted to some extent. The more traditional way of cryo-EM structure determination is using 2D projections of molecules in various directions to achieve 3D reconstruction, based on the rationales DeRosier and Klug built with negative stained samples (De Rosier and Klug, 1968) (Fig. 2).

The basis of cryo-EM 3D reconstruction is the theorem named projection-slice theorem, or central/Fourier slice theorem. The theorem states that the Fourier transform of a 2D projection of a 3D object in real space is equivalent to a central 2D slice of the 3D Fourier transform of that object. The real-space projection direction is orthogonal to the slice. TEM images represent the 2D projections of molecules in the sample. Multiple copies of same molecules in the vitrified sample are preserved in various directions. Considering these facts synthetically, if the directions of the 2D projections are known, 2D Fourier slices of these projections can be positioned back into the 3D Fourier space; thus, the reconstruction of the origin molecular structure can be done by computing the inverse Fourier transform. The resolution of reconstructed results can be improved through performing multiple iterations. With sufficient number of images containing high resolution information that are accurately classified and aligned, 3D structure at atomic resolution can be obtained by single particle reconstruction.

The problem of low signal-to-noise ratio (SNR) caused by low electron dose imaging has accompanied cryo-EM technology since its birth. The loss of particle spatial orientation and position information including three Euler angles, two in-plane positional parameters as well as the defocus value also hinders the reconstruction. Even though theoretical assessment suggests that single particle analysis may reach atomic resolution for molecules with a molecular weight of around 100 kDa, the reality has been far from satisfactory for a long period (Bai et al., 2015).

Tremendous efforts have been made in aspects of both hardware and algorithm. Commonly used optimization methods for commercialized EM include applying constant-power electromagnetic lenses, using field emission gun (FEG) as electron source for a better parallel illumination, adopting high vacuum, and employing better computer control for microscope tuning and data acquisition (Cheng, 2015). The most revolutionary progress for instrumentation in recent years is the advent of direct electron detection devices (DDDs). DDDs produce significantly better images than traditional film or charge-coupled devices (CCDs). DDD cameras no longer converts electronic signals into optical signals but detects electrons directly. Individual electrons are identified (Cheng, 2015; Kuijper et al., 2015; Nogales and Scheres, 2015). With the high-enough SNR and location of each electron event being determined precisely enough, DDD gives much higher detective quantum efficiency (DQE) (McMullan et al., 2014). If the arrival point of individual electrons can be determined to subpixel accuracy, DDDs might be used in super-resolution mode beyond Nyquist cut-off frequency (Kuijper et al., 2015; McMullan et al., 2016).

Another important feature of DDD cameras is their fast frame readout rate. This allows the total electron dose used to image biological samples to be split into multiple frames, making the final output into subframe-motion-correctable movies (Bai et al., 2015; Cheng, 2015). Drift correction of these frames before averaging can eliminate the beam-induced image blurring.

Many advances in image processing methods have also been made. After the concept of single particle analysis was established in 1970s, multivariate statistical analysis was introduced into the particle classification process in 1981 (van Heel and Frank, 1981). In the same year, modular software pioneered by Joachim Frank and Marin van Heel for electron image processing emerged (Frank et al., 1981; van Heel and Keegstra, 1981). Then in 1987, ab initio methods for projection angle determination termed random conical tilt and angular reconstitution (common lines) were proposed (Radermacher et al., 1987). Projection matching and angular refinement method were set up in 1994 (Penczek et al., 1994), followed by the adhibition of maximum-likelihood approach in 1998 (Sigworth, 1998). Based on these sound foundations, more algorithmic optimization methods are proposed, such as the introduction of the empirical Bayesian approach and the gold-standard approach (Scheres, 2012a, b; Scheres and Chen, 2012), stochastic gradient descent (SGD) and branch-and-bound maximum likelihood optimization algorithms (Punjani et al., 2017), the sequential Monte Carlo method based particle-filter algorithm (Hu et al., 2018), and different approaches used for automated particle picking (Nicholson and Glaeser, 2001; Adiga et al., 2004; Ogura and Sato, 2004; Wong et al., 2004; Langlois et al., 2014; Wang et al., 2016; Al-Azzawi et al., 2019b, a; Wagner et al., 2019). These explorations eventually led to the birth of the widely used semi-automated 3D reconstruction software.

Another thing worth noting is the motion correction of the raw data collected from cryo-EM equipped with DDD cameras. The inevitable movement of molecules during the data acquisition reduces the overall quality of the photographs and the final resolution of the reconstruction obtained from the data. To better understand and deal with beam-induced motion is one of the most challenging physical problems for cryo-EM. Beam-induced sample motion can be divided into two components, uniform whole-frame motion and idiosyncratic local motion (Brilot et al., 2012; Li et al., 2013). In 2013, an algorithm for correcting relative motion between subframes was published, in which measurements of image shifts between all frames are performed to calculate least-squares estimates of relative shifts between adjacent frames. It provides an effective correction of whole-frame motions with sufficient accuracy for near-atomic-resolution 3D reconstructions (Li et al., 2013; Zheng et al., 2017). Individual particle tracking or local motion correction methods are proposed to simulate and estimate particle trajectories and cumulative beam-induced damage, improving the resolution of 3D reconstruction (Bai et al., 2013; Scheres, 2014; Rubinstein and Brubaker, 2015; Scheres, 2016; Zheng et al., 2017; Zivanov et al., 2019).

The craft of sample preparation is another fast-developing area. The invention of semi-automated sample preparation robots is a great convenience; however, its stability and repeatability still need to be improved. Other promising technologies include the preparation of graphene supporting film (Russo and Passmore, 2016), whisker-assisted blotting (Razinkov et al., 2016; Frank, 2017a), and spraying-plunging method (Feng et al., 2017).

## Cryo-EM structures of the mammalian respirasomes

The 3D structure of respirasome has been studied with EM at low resolutions from 33 to 18 Å (Schafer et al., 2006; Schafer et al., 2007; Althoff et al., 2011; Dudkina et al., 2011). The emergence of new technologies has triggered an upsurge in the use of cryo-EM in structural biology. This wave swept through broad and diverse areas, including the structural research of the ETC. In 2014, first modern cryo-EM structure of bovine complex I was reported (Vinothkumar et al., 2014). 2016 is a momentous year for the study of respirasomes, with near-atomic-resolution structures of integral mammalian CI and SCI_1_III_2_IV_1_ being reported in succession.

The publication of the 4.2 Å structure of the first integral bovine-heart CI opened the prelude to these exciting results (Zhu et al., 2016). In the immediate aftermath, we and Sazanov’s group reported the architectures of porcine and ovine respirasome SCI_1_III_2_IV_1_ at 5.4 Å and 5.8 Å, respectively (Gu et al., 2016; Letts et al., 2016). The intact mammalian CI structure at 3.6 Å derived from SCI_1_III_2_IV_1_, which is the first atomic resolution structure of CI, was also obtained (Wu et al., 2016) shortly afterwards. In 2017, our group pushed the resolutions of porcine and human SCI_1_III_2_IV_1_ to 4.0 Å and 3.9 Å (Guo et al., 2017). Our firstly obtained human respirasome structure provided the most accurate and detailed map of mutations related to severe mitochondrial malfunction diseases, including Alzheimer’s disease, Huntington’s disease, Parkinson’s disease, Friedreich’s ataxia and so on. This map of mutations could help drug-development and provide reference for prenatal diagnose of inheritable genetic diseases.

Until the year 2019, structures of mammalian SCI_1_III_2_IV_1_ in different states from various species have been deposited into PDB, laying the foundation for further discussion of the functional mechanism (Fig. 1A and 1B). The mammalian respiratory SCI_1_III_2_IV_1_ has a dimension of around 300 Å in length and 190 Å in height. The L-shaped CI is composed of a transmembrane arm and a hydrophilic arm (matrix arm). The TM arm is located in the mitochondrial IM, bending slightly inward with its concave surface interacting with CIII_2_. CIV is positioned on the concave surface formed by CIII_2_ and the distal end of the TM arm of CI. According to our structures, the final human SCI_1_III_2_IV_1_ contains 45 subunits from CI, 21 subunits from CIII and 14 subunits from CIV, and possesses 133 transmembrane helices (TMHs), with 78, 26 and 29 TMHs from CI, CIII and CIV, respectively.

The interactions between CI and CIII in mammalian SCI_1_III_2_IV_1_ mainly take place in two regions: the first where the NDUFA11 and NDUFB4 subunits of CI interact with UQCRQ subunit of CIII, and the second where the NDUFB9 and NDUFB4 subunits of CI interact with the UQCRC1, UQCRFS1 subunits of CIII (Gu et al., 2016; Letts et al., 2016; Sousa et al., 2016; Wu et al., 2016; Guo et al., 2017; Milenkovic et al., 2017; Hirst, 2018).

The cytochrome c oxidase subunit VIIa polypeptide 2-like protein (Cox7A2L), also known as supercomplex assembly factor 1 (SCAF1), is proposed to be essential for the interaction between CIII and CIV but does not have significant impact on the assembly or function of the respirasome (Ikeda et al., 2013; Lapuente-Brun et al., 2013; Mourier et al., 2014; Perez-Perez et al., 2016). The Cox7A2 subunit in our human CIV structure may consist with results reported in mouse strain C57BL/6 that mutation of its long isoform Cox7A2L impairs the formation of the SCIII_2_IV_1_, while respirasomes can exists in fully assembled form in mice expressing both long and short forms of Cox7A2L (Mourier et al., 2014; Cogliati et al., 2016; Williams et al., 2016). Previous research also indicate that Cox7A1/A2 and Cox6A1/A2 are tissue-specific expressed subunits, with Cox7A1 and Cox6A2 promoting CIV dimerization (Cogliati et al., 2016; Milenkovic et al., 2017). The Cox7A1 and Cox6A2 subunits in our monomeric human CIV structure are replaced by Cox7A2 and Cox6A1 subunits, which is consistent with the previous conclusion (Zong et al., 2018b).

Phospholipids play an important role in maintaining the structure and function of respirasomes. The formation, stabilization and function of respirasomes are impacted by the lipid composition of the IMM. The most discussed phospholipids include phosphatidylcholine (PC), phosphatidylethanolamine (PE), and cardiolipin (CDL). The phospholipids identified in the published structures of respirasomes confirmed their importance in stabilizing respirasomes and helping respirasomes complete conformational change and fulfill their functions (Zhang et al., 2002; Enríquez, 2016; Letts et al., 2016; Wu et al., 2016; Guo et al., 2017; Milenkovic et al., 2017) (Fig. 1C).

The mechanism underlying the conformational change between “active” and “deactive” states of CI has been discussed following the reporting of CI structures (Fiedorczuk et al., 2016; Agip et al., 2018; Blaza et al., 2018; Parey et al., 2018). The conserved features of CI in different species including π-bulges, interrupted TMHs and charged residues in the membrane plane could form the structural basis for the function of CI. The deactivation of CI may be related to the structural disorder in the Q binding site and the conformational change of the TMH3 in ND6 (Agip et al., 2019; Letts et al., 2019).

## CIII and CIV, news in olds

Mammalian CIII_2_ has long been regarded as a homodimer consisting of 22 subunits in total in the past structural studies. But the full length N-terminal processed peptide (UQCRFS1N) of the iron-sulfur Rieske protein (UQCRFS1) subunit was not assigned in all of these structures. By rebuilding the high-resolution crystal structures of bovine and chicken CIII and analyzing the reconstructed density map of cryo-EM, we draw a new conclusion that the two 10-subuint CIII protomers are linked by a single UQCRFS1N molecule. The N-terminal segment and the C-terminal segment of one UQCRFS1N molecule bind with each CIII protomer respectively, with the two protomers being identical. Both protomers of CIII_2_ are able to bind with CI, and consequently our reconstructed cryo-EM density map of SCI_1_III_2_IV_1_ show a mixed feature of the two possibilities (Zong et al., 2018a). TTC19 protein is reported to be essential for removing UQCRFS1N from the full-length UQCRFS1 subunit. In mitochondria lacking TTC19, CIII_2_ contains two full-length UQCRFS1 with two UQCRFS1N segments. Experimental results showed that the native molecular mass of CIII_2_ from Ttc19^−/−^ knockout mouse is slightly higher than that of CIII_2_ from wild type animals, which may indicate it to be consistent with our conclusion (Bottani et al., 2017).

NDUFA4 was originally considered as a subunit of CI, and although there was still controversy (Kadenbach, 2017), further studies indicated that this protein should belong to CIV (Balsa et al., 2012; Pitceathly and Taanman, 2018). However, NDUFA4 was never found in current CIV crystal structures that present as homodimers. In SCI_1_III_2_IV_1_, CIV rolls around the tip of CI. The state when CIV is relatively closer to CI is more stable than the state when CIV is approaching CIII, and the contents of particles at these two states are basically the same. This means that if we mix up all kinds of particles, the auto-refine of complex IV by adding a soft mask would fail due to the excessive differences between these two parts. If we only use one conformation, the resolution of the SCI_1_III_2_IV_1_ would be too low to provide a sufficient accuracy for local optimization. Therefore, we put all particles together and refine it to the highest resolution. After adding a soft mask to CIV, we classify the particles into different conformations without performing particle alignment. In the last step, the class with clear structure and the highest proportion of particles was chosen and refined. Eventually the reconstructed density map of human CIV at a resolution of 3.3 Å was obtained, where we found the precise location of NDUFA4 subunit.

NDUFA4 may hamper the formation of CIV dimer by binding to the dimeric interface of the crystal structures of CIV. The TMH of NDUFA4 in CIV monomer clash with that of Cox6A2 in another protomer of CIV dimer. BN-PAGE analysis in different species with different detergents and 2D crystal data in lipid bilayer indicate that CIVs may exist as monomers in membranes, while NDUFA4 subunit is important for the formation of CIV (Wittig et al., 2006a; Balsa et al., 2012; Osuda et al., 2016). We concluded that NDUFA4 is a subunit, not an assembly factor, of CIV, and CIVs are more likely to exist as monomers in native state.

## Find a way in the maze for electrons

One of the most important goals for in studying the structure of ETC complexes and supercomplexes is to understand the functional mechanism of the respiratory chain. Respirasome was supposed to enhance the electron transporting efficiency through substrate channeling (Vartak et al., 2013; Genova and Lenaz, 2014; Lobo-Jarne and Ugalde, 2018). The interaction between CI and CIII may contribute to the functional division of Q pool (Heron et al., 1978; Ragan and Heron, 1978; Schägger and Pfeiffer, 2000; Bianchi et al., 2004) and is also conducive to the modulation of ROS generation by isolating reactive intermediates (Wang et al., 2010; Maranzana et al., 2013; Letts et al., 2016). Assembly into supercomplexes also conduces to the stabilization and activation of CI (Lamantea et al., 2002; Acin-Perez et al., 2004; Schagger et al., 2004; Vempati et al., 2009).

Biochemical experimental results have indicated the functionally relevant association between CI and CIII, and the CIII-CIV interaction also induces the possibility of a cyt c pool. Therefore, a widely held opinion regards the enhanced catalysis of the respirasome to be achieved by the substrate channeling translocating intermediate substrates from complex to complex and the assembly of ETC complexes to be the result of the functional segmentation of Q pools and cyt c pools (Genova and Lenaz, 2011; Enríquez, 2016; Hirst, 2018).

Evidence have been provided against the existence of a single Q pool (Lenaz and Genova, 2012). Further spectroscopic and kinetic analysis also show the existing contradictions with both the proposals of a single or two fully independent pools (Q_NADH_ pool and Q_FAD_ pool) (Blaza et al., 2014). Recent studies pointed out that the QH_2_ from CI is reoxidized more rapidly by added AOX outside the SC than by CIII inside the SC, strongly questioning the existence of the substrate channel and the segmented Q pool (Milenkovic et al., 2017; Fedor and Hirst, 2018; Hirst, 2018). Meanwhile, no confining protein structures are found between CI and CIII that could guide the diffusion of Q/QH_2_ (Gu et al., 2016; Letts et al., 2016; Sousa et al., 2016; Wu et al., 2016; Guo et al., 2017). Scientists also suggest that a strictly restrained channel and Q pool may increase the risk of the system resisting the dysfunction of ETC complexes (Hirst, 2018). Additionally, the existence of functionally segmented cyt c pool is also questioned (Trouillard et al., 2011).

**Figure 3:**
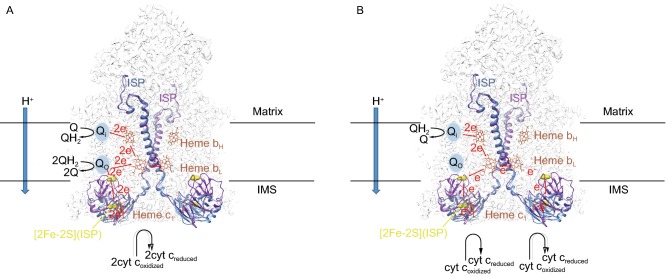
**Mechanisms of CIII**_**2**_. (A) Diagram of Q cycle theory. The ISP proteins and catalytic centers are shown in different colors. The ISP subunit colored in blue (PDB ID: 5XTE, also called as “c_1_ state”) and the ISP subunit colored in purple (PDB ID: 3H1I, also called as “b state”) (Zhang et al., 1998) indicate the conformational change between two states of CIII during electron transportation. The black dotted lines represent the mitochondrial inner membrane. The black arrows represent the change of substrates. The blue arrow indicates the proton translocation direction. The red dotted lines indicates the electron flow in CIII dimer. The light blue circles represent the Q-binding site Q_i_ and Q_o_. (B) Diagram of the mechanism raised in our paper. The labels are consist with Fig. 3A

The prevailing view of the electron transfer process in CIII is described by Q cycle theory first proposed by Peter Mitchell (Mitchell, 1975a, b; Crofts et al., 1983). This theory indicates that the oxidation of QH_2_ at the Q_o_ site is a bifurcated reaction. In the reaction, QH_2_ binds to Q_o_ site, while Q binds to Q_i_ site. Two electrons obtained from the oxidation of QH_2_ flow to ISP and heme b_L_, respectively. ISP accept one electron and pass it on to cytochrome c_1_, then to cyt c. The other electron transports along the chain heme b_L_–heme b_H_–Q (bound to Q_i_ site). Two cycles are needed to reduce the Q bound at Q_i_ site to QH_2_ (Pietras et al., 2016; Sousa et al., 2018) (Fig. 3A).

Despite that the Q cycle theory has been popular for a long time, there is still no high-resolution structure or reliable data to support the claim that the Q_o_ site is a functional QH_2_-binding site (Pietras et al., 2016). The difficulty in identification of the intermediate states of Q_o_ site catalysis, as well as the necessity of avoiding semiquinone- mediated short circuits has also left a high degree of freedom for mechanistic interpretation (Osyczka et al., 2004; Osyczka et al., 2005; Pietras et al., 2016).

After analyzing the structure of CIII, we propose a new mechanism for the electron transfer process of CIII. In our model, QH_2_ released from CI binds to Q_i_ site of CIII. Heme b_H_ accepts one electron from QH_2_ and passes it to heme b_L_, while accepting the other electron simultaneously. The oxidized Q is released from Q_i_ site. Heme b_L_ accepts the first electron and transfers it to the heme b_L_ in the other CIII monomer, enabling the first heme b_L_ to accept the other electron from heme b_H_. Heme b_L_ transfers the electrons to ISP, and then to cyt c_1_, and eventually cyt c. In this way, the accumulation of semi-quinone radical is reduced, thus minimizing the generation of ROS. At the same time, as the electron transportation between two heme b_L_ molecules could be faster than the conformational change of ISP, the electron transfer between heme b_L_ molecules could occur earlier in our model (Wu et al., 2016; Guo et al., 2017) (Fig. 3B). During this process, two protons dissociated from QH_2_ are translocated to the IMS using the redox energy released by electron transfer.

Our model provides an explanation to the phenomenon that the addition of myxothiazol, an inhibitor bound at Q_o_ site, could stimulate generation of superoxide at Q_o_ site (Starkov and Fiskum, 2001; Muller et al., 2002). After two electrons are transferred from QH_2_ at Q_i_ site to two heme b_L_, conformational change of ISP becomes the rate limiting step. The binding of myxothiazol even further slow down the conformational change of ISP, causing electrons backing up at heme b_L_, becoming the source of superoxide.

The asymmetric Q binding densities in CIII segment of SCI_1_III_2_ may indicate a more complicated functional mechanism (Letts et al., 2019). More research are needed to clarify the mechanism of electron transportation in individual ETC complexes and the respirasome as a whole, which could include analyzing atomic structures of SCs at more states and conducting related biophysical and biochemical experiments. It is possible that EPR could help us understand the pathway of electron transfer in the respirasome (Ohnishi et al., 2012; Pietras et al., 2016; Wright et al., 2016).

## Megacomplex, calling for technology progress

The concept of megacomplex has long been brought along by EM/ET analyses. In the early studies, the possibility of the existence of dimeric CI was proposed, accompanied by the anticipation of finding convincing evidence for a respiratory string composed of CI, CIII and CIV (Allen et al., 1989; Nubel et al., 2009; Wittig and Schagger, 2009; Strecker et al., 2010). Accordingly, models of respiratory strings with different basic units and conjunction modes were raised (Wittig et al., 2006b; Bultema et al., 2009; Vonck, 2012; Letts et al., 2016). MCI_2_III_2_IV_2_ was first supposed to be the block of a modality of respiratory string in our results published in 2016 (Wu et al., 2016), inspired by the identification of the supramolecular assembly form I_2_III_2_ in potato (Bultema et al., 2009).

During our study of the respirasome, we noticed the existence of higher-order assemblies of ETC complexes as have been indicated by previous investigations. First, we detected high-molecular-weight bands above SCI_1_III_2_IV_1_ in BN-PAGE analysis. The results of mass spectrometry analysis suggest that the main components of these bands are subunits of the ETC complexes, particularly CI, CIII and CIV. Negative stained samples were prepared for EM examination. A minor population of particles with an circular arrangement were detected. These findings led to our proposal of a higher oligomeric state named megacomplex- I_2_III_2_IV_2_ (MCI_2_III_2_IV_2_) published in 2016. We assumed this assembly form as a structure with dimeric CIII located in the center, surrounded by two CIs with their membrane arms oriented perpendicularly to the 2-fold axis of CIII_2_. Two CIVs were anchored by the distal end of each CI membrane arm and CIII_2_. The MCI_2_III_2_IV_2_ contains 139 subunits in total (45 from each CI, 14 from each CIV, and 21 from CIII_2_), with a height of ~220 Å, width of ~280 Å, and length of ~300 Å (Guo et al., 2017).

Through further analysis of large amounts of cryo-EM data, we obtained the 3D reconstruction architecture of MCI_2_III_2_IV_2_ at a resolution of 17.4 Å in 2017 with about 8,600 particles out of 1.18 million (Guo et al., 2017) (Fig. 1D). These results provoked intense debates immediately. Skeptical opinions center around the point that MCI_2_III_2_IV_2_ might be an artifact caused by the usage of specific detergent (digitonin) or the tendentious collection of nonspecific particles, because of the extremely low relative content and the lack of in situ detection or biochemical experimental evidence. However, NBT staining of our BN-PAGE result indeed detected a catalytically active band above the band of respirasome (Guo et al., 2017), which we believe to be the proof of existing higher organization form than respirasome.

In our following study, we improved the relative concentration of MCI_2_III_2_IV_2_ and pushed the reconstruction results to a better resolution where secondary structures can be clearly visualized. Different initial models and unsupervised ab initio reconstructions are tried to avoid model-bias effect. MCI_2_III_2_IV_2_ are found both in porcine and human derived mitochondrial extracts. According to our megacomplex structure with higher resolution, we can even detect direct interactions between CIII and two CIs, and two CIVs bind to CIII in a different pattern from respirasome (unpublished data). However, these progresses did not dispel all the suspicion, mostly because no megacomplex was resolved from cryo-ET images. We must admit the relative ratio of MCI_2_III_2_IV_2_ is quite low, so it could be difficult to detect MCI_2_III_2_IV_2_ from a multiple of mitochondrial slices. In contrast, during single particle data processing, proteins from mitochondria in various states can be extracted, so the chance of detecting MCI_2_III_2_IV_2_ is appearently higher. Therefore, technology progress of cryo-ET is in urgent need to reconstruct supramolecular assemblies in mitochondria extracted from different stages of cell division cycle.

Cryo-ET and subtomogram averaging results of mitochondrial membranes strengthened the skepticism about the existence of the ubiquitous respiratory strings, suggesting that SCI_1_III_2_ is the most conserved structure across different species (Davies et al., 2011; Davies et al., 2018). In contrast, however, the recently published structure of SCIII_2_IV_1–2_ from Mycobacterium smegmatis and yeast may indicate the functional necessity of a cytochrome subunit bridged electron transfer path for ETC complexes in prokaryotic cells (Gong et al., 2018; Wiseman et al., 2018; Hartley et al., 2019; Rathore et al., 2019).

As a powerful tool for in situ reconstruction, cryo-ET is one of the most promising technology that might be able to extricate us from the current predicament. However, cryo-ET is facing the dilemma once faced by cryo-EM single particle analysis—the bottleneck of limited resolution, which is a comprehensive result of manifold causes. For cryo-ET, effective methods still need to be derived in areas including sample preparation, determination of the area to be detected, phase plate performance, motion correction and tilt series alignment, CTF estimation, treatment for the loss of information at high tilt angles, image processing algorithm and many more. Subtomogram averaging provides a scheme to balance the advantages of both single particle analysis and traditional cryo-tomography methods (Wan and Briggs, 2016; Leigh et al., 2019; Schur, 2019). With the application of newly developed 3D-CTF estimation algorithm, the resolution of subtomogram averaging reconstruction could reach 3.4 Å (Turonova et al., 2017). This methodology is largely a variant of single particle analysis, thus the large-scale in situ reconstruction at high resolution still needs the invention of new techniques and new ideas.

Time-resolved cryo-EM is also a possible developing trend depending on better classification methods and dynamic simulation algorithms (White et al., 2003; Fu et al., 2016; Frank, 2017b). As the star technology gaining great attention, cryo-EM/ET still has tremendous potential to be explored.

## Conclusions and perspectives

The curiosity into respiration encapsulates people’s desire for exploring the mysteries of life. Through the efforts of scientists over the past century, features of the molecular machineries that are responsible for this physiological process have become gradually known to us. The rapid development in cryo-EM technology provides us with a powerful tool to break through the bottleneck of analyzing the atomic structure of macromolecular protein complexes, pushing the research field of respirasome forward into a new stage. High- resolution structures of eukaryotic respiratory supercomplex I_1_III_2_IV_1_ and III_2_IV_2_ have been acquired successively. New features of mammalian ETC complex III and IV have also come into view. The discovery of MCI_2_III_2_IV_2_ may provide a new possibility to the study of the electron transfer mechanism. With new theoretical arguments and experimental results emerging, widely accepted theories are starting to be challenged. All of these results together could help us clarify the functional mechanism of the respirasome, which is of great significance to elucidating the pathogenesis of mitochondrial diseases and finding the treatment methods of mitochondrial diseases.

**Figure 4:**
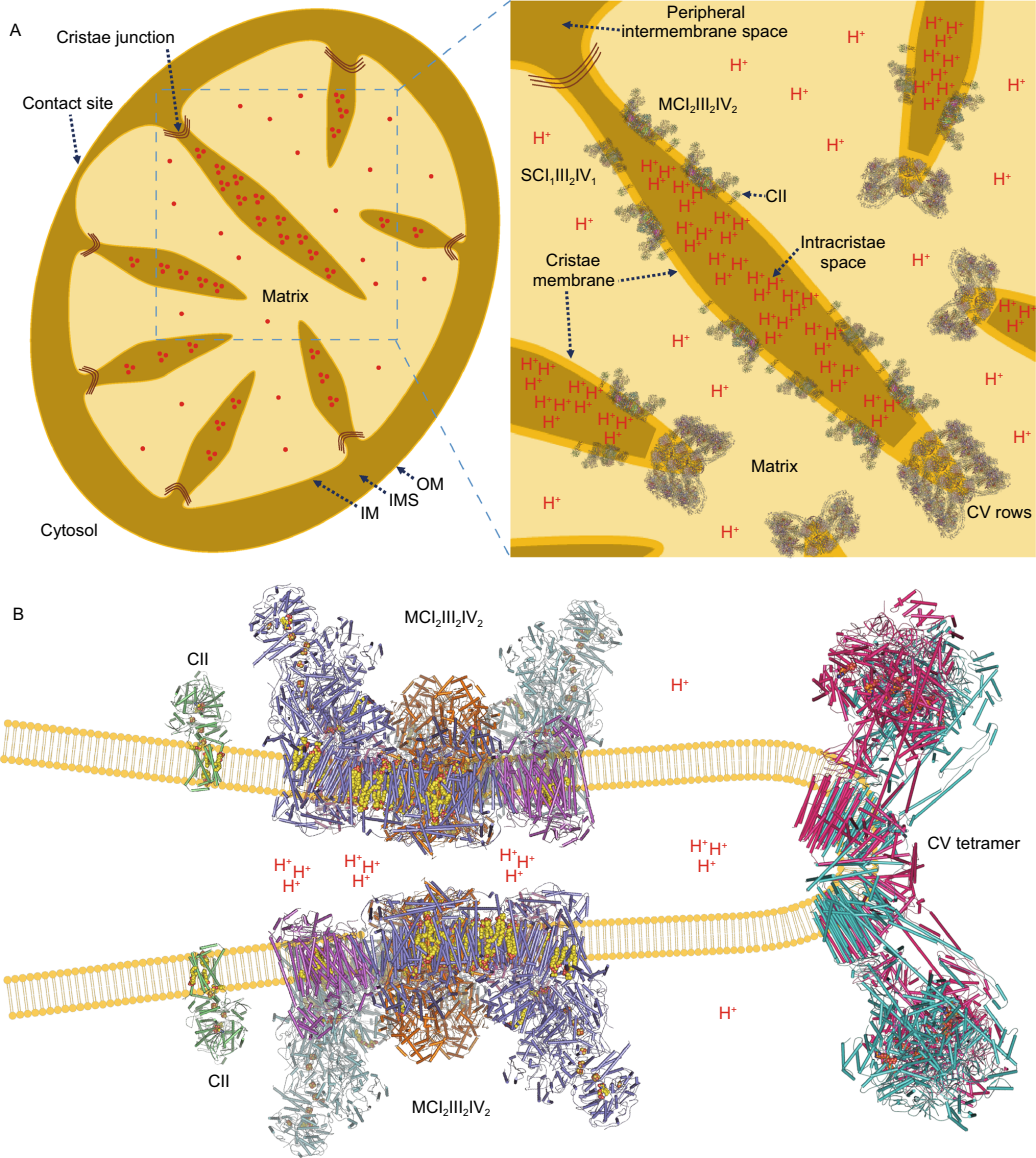
**Orgnization and distribution of mammalian ETC complexes in mitochondria.** (A) Diagram of mitochondrial internal morphology (left). The part in the blue dotted frame is enlarged (right) with the simulated organization and distribution of ETC complexes shown. The proton concentration gradient is represented with red notes. PDB ID of CII: 1ZOY. PDB ID of MCI_2_III_2_IV_2_: 5XTI. PDB ID of SCI_1_III_2_IV_1_: 5XTH. PDB ID of CV: 6J5K. (B) Distribution of CII, MCI_2_III_2_IV_2_ and CV tetramer on mitochondrial cristae. Cofactors are shown in spheres. The structures used are the same with Fig. 4A

ETCs are the most abundant protein within mitochondrial inner membrane, and they are closely related to the maintaining of cristae shape. Taken our structures and the in situ cryo-ET images together, it is now widely accepted that the CV oligomers sit on the tip of cristae ridges, while respiratory chain complexes are located on the relatively flat cristae plane, whether assemble into supercomplex (even megacomplex) or stay alone. Protons are enriched in the intracristae space, especially at the cristae tips. This arrangement of ETCs could be most efficient in energy conversion (Fig. 4A and 4B), For complexes I, II, III, IV, and V in bovine heart mitochondria, a ratio (1.1 ± 0.2) : (1.3 ± 0.1) : 3 : (6.7 ± 0.8) : (3.5 ± 0.2) was determined (Schagger and Pfeiffer, 2001). Appearantly, the ratio of CIV is much higher than CI and CIII, so the binding pattern of CIV could be either with CI, CIII, or staying alone. Actually, we even identified some respirasome particles containing more than one copy of CIV.

However, the recent debates over the new proposals indicate that much more evidence are still needed to dispel the doubts. In situ reconstruction of respirasomes under different cell conditions might provide a break point to the current situation. In situ and time- resolution reconstruction are the developing directions of cryo-EM/ET that have received wide attention. For cryo-ET, resolution of the 3D reconstruction is still the most prominent limitation for its application. The advances in super-resolution light microscopy may also be useful for dynamic research of respirasomes. In addition, more biochemical and biophysical experiments are also needed to help clarify the speculations.

The history for scientists to get to know respirasome is a combination of technological development and theoretical innovation, which also holds true in the broad scientific world. With the combined effort of all scientists in this field, we expect that one day the whole picture of respiration could be presented to us.
